# Cardiac magnetic resonance left ventricular filling pressure is linked to symptoms, signs and prognosis in heart failure

**DOI:** 10.1002/ehf2.14499

**Published:** 2023-08-19

**Authors:** Ciaran Grafton‐Clarke, Pankaj Garg, Andrew J. Swift, Samer Alabed, Ross Thomson, Nay Aung, Bradley Chambers, Joel Klassen, Eylem Levelt, Jonathan Farley, John P. Greenwood, Sven Plein, Peter P. Swoboda

**Affiliations:** ^1^ Norwich Medical School University of East Anglia Norwich Research Park Norwich NR4 7UQ UK; ^2^ Norfolk and Norwich University Hospitals NHS Foundation Trust Norfolk UK; ^3^ Department of Infection, Immunity and Cardiovascular Disease University of Sheffield Medical School and Sheffield Teaching Hospitals NHS Trust Sheffield UK; ^4^ Department of Clinical Radiology Sheffield Teaching Hospitals NHS Foundation Trust Sheffield UK; ^5^ William Harvey Research Institute, NIHR Barts Biomedical Research Centre Queen Mary University of London London UK; ^6^ Barts Heart Centre St Bartholomew's Hospital, Barts NHS Trust London UK; ^7^ Leeds Institute of Cardiovascular and Metabolic Medicine University of Leeds Leeds UK

**Keywords:** Left ventricular filling pressure, Heart failure, Cardiovascular magnetic resonance, Heart failure with reduced ejection fraction, Heart failure with preserved ejection fraction

## Abstract

**Aims:**

Left ventricular filling pressure (LVFP) can be estimated from cardiovascular magnetic resonance (CMR). We aimed to investigate whether CMR‐derived LVFP is associated with signs, symptoms, and prognosis in patients with recently diagnosed heart failure (HF).

**Methods and results:**

This study recruited 454 patients diagnosed with HF who underwent same‐day CMR and clinical assessment between February 2018 and January 2020. CMR‐derived LVFP was calculated, as previously, from long‐ and short‐axis cines. CMR‐derived LVFP association with symptoms and signs of HF was investigated. Patients were followed for median 2.9 years (interquartile range 1.5–3.6 years) for major adverse cardiovascular events (MACE), defined as the composite of cardiovascular death, HF hospitalization, non‐fatal stroke, and non‐fatal myocardial infarction. The mean age was 62 ± 13 years, 36% were female (*n* = 163), and 30% (*n* = 135) had raised LVFP. Forty‐seven per cent of patients had an ejection fraction < 40% during CMR assessment. Patients with raised LVFP were more likely to have pleural effusions [hazard ratio (HR) 3.2, *P* = 0.003], orthopnoea (HR 2.0, *P* = 0.008), lower limb oedema (HR 1.7, *P* = 0.04), and breathlessness (HR 1.7, *P* = 0.01). Raised CMR‐derived LVFP was associated with a four‐fold risk of HF hospitalization (HR 4.0, *P* < 0.0001) and a three‐fold risk of MACE (HR 3.1, *P* < 0.0001). In the multivariable model, raised CMR‐derived LVFP was independently associated with HF hospitalization (adjusted HR 3.8, *P* = 0.0001) and MACE (adjusted HR 3.0, *P* = 0.0001).

**Conclusions:**

Raised CMR‐derived LVFP is strongly associated with symptoms and signs of HF. In addition, raised CMR‐derived LVFP is independently associated with subsequent HF hospitalization and MACE.

## Introduction

The number of people living with heart failure (HF) has been increasing. This is the result of an ageing population, improved survival after diagnosis, and recognition of HF with preserved ejection fraction (EF) as a clinically important phenotype.^1^ Cardiovascular magnetic resonance (CMR) imaging is an ever‐evolving diagnostic tool to guide clinical management and therapeutic decision‐making in people with suspected or proven HF.^2^, ^3^ CMR can provide deep insight into the aetiology of HF through its capability to characterize the myocardium, particularly by late gadolinium enhancement (LGE) imaging. CMR is the reference standard for assessing left ventricular ejection fraction (LVEF), which is a prognostic marker in HF.^4^ Although, in clinical practice, LVEF has a relatively small association with survival, factors such as age and comorbidities are more important.^3^

Raised left ventricular filling pressure (LVFP) is also an important prognostic factor that can be identified clinically once the person develops evidence of jugular venous distension or peripheral oedema.^5^ LVFP is best measured by right heart catheterization (RHC), although its use in clinical practice is decreasing. One of the key goals of cardiovascular imaging is to identify people with increased LVFP before they develop overt congestion and decompensation without the need for invasive assessment.

In a recent observational study involving 835 participants, a CMR‐derived model to estimate LVFP was developed and validated by RHC performed within 24 h.^6^ The CMR‐derived LVFP model originates from a single‐centre study and needs to be validated in external cohorts of subjects with HF.^7^

This study aimed to test CMR‐derived LVFP in a real‐world population of patients undergoing CMR to identify the aetiology of HF. The primary objective was to evaluate whether CMR‐derived LVFP added independent prognostication over established CMR parameters, specifically LVEF and the presence of ischaemic scar. The secondary objective was to investigate the relationship between CMR‐derived LVFP and the signs and symptoms of HF.

## Methods

Between February 2018 and January 2020, adult patients seen in cardiology clinic with a diagnosis of HF in the preceding 12 months, according to the European Society of Cardiology Heart Failure guidelines, were prospectively recruited.^3^ These criteria include the presence of at least one symptom of HF (e.g. breathlessness), one sign of HF (e.g. peripheral oedema), and objective evidence of cardiac dysfunction (i.e. reduced EF on echocardiography). Those eligible proceeded to CMR and same‐day clinical assessment.

Participants were not eligible for inclusion if they had a known history of coronary artery disease (at least one of the following: stenosis > 70% during invasive angiography, known myocardial infarction, previous percutaneous coronary intervention, or coronary artery bypass grafting), symptoms consistent with angina pectoris, hypertrophic cardiomyopathy, or congenital heart disease. Aligning with a pragmatic approach to participant inclusion, those with evidence of ischaemia on CMR were not retrospectively excluded, as, in the real‐world setting, CMR has utility in exploring the cause of HF. Those with acute pathologies were excluded, such as myocarditis, acute renal impairment, or any contraindication to CMR or gadolinium‐based contrast agents.

Participant outcomes were evaluated by reviewing electronic hospital records for major adverse cardiovascular events (MACE) and hospitalization due to HF. MACE was defined as the composite of cardiovascular death, HF hospitalization, non‐fatal stroke, and non‐fatal myocardial infarction.

All participants provided written informed consent to participate in the study. The study protocol was approved by the National Research Ethics Service (17/YH/0300) in the United Kingdom. The investigation conforms with the principles outlined in the Declaration of Helsinki.^8^

### Study cohort

Participants underwent a clinical assessment on the day of their CMR appointment. This included determining New York Heart Association (NYHA) functional class, evaluating for cardiovascular disease risk factors, and recording comorbid conditions and current medications. Participants were asked about specific HF symptoms, including breathlessness and orthopnoea. All participants were physically examined, which included an assessment of lower limb peripheral oedema. Blood was drawn at the time of intravenous cannulation and serum frozen at −70°C. N‐terminal pro‐B‐type natriuretic peptide (NT‐proBNP) was measured from defrosted serum in one batch.

### Cardiovascular magnetic resonance imaging

All CMR studies were undertaken on a 3 T system (Siemens Magnetom Prisma, Erlangen, Germany). Participants were instructed to abstain from caffeine for 24 h before the study. The protocol included cine imaging, native and post‐contrast T1 mapping, stress and rest perfusion, and LGE imaging. Adenosine was infused at 140–210 mcg/kg/min (depending on the haemodynamic response) for pharmacological stress.^9^ Perfusion images were acquired in three short‐axis slices using a T1‐weighted saturation recovery gradient echo sequence after administering gadolinium‐based contrast. Late gadolinium imaging was performed 10–15 min after the final contrast injection, maintaining the same long‐ and short‐axis slice positions as in cine imaging, and employing a segmented inversion‐recovery gradient echo sequence.

If it was unclear whether enhancement on bright blood LGE was seen, a dark blood LGE was acquired for further clarification. Bilaterally, the presence of fluid on black blood axial imaging within the costophrenic angle was used as the criteria for pleural effusion.

### Cardiovascular magnetic resonance image analysis

Measurement of cardiac volumes and the presence of LGE were assessed using cvi42 software (Circle Cardiovascular Imaging, Calgary, Canada). Left ventricular mass (LVM) was calculated by the summation of discs method at end‐diastole. Left atrial volume (LAV) was calculated by the area length method from long‐axis cines prior to atrial contraction. LGE was reported if enhancement was identified on two orthogonal planes or, where available, on both bright and dark blood LGE images. Ischaemic LGE was defined as involving the subendocardium in a typical coronary distribution. Non‐ischaemic LGE was defined if enhancement did not involve the subendocardium. Inducible ischaemia was defined if a visual perfusion defect was present in one segment during stress testing but not at rest or matching an infarct on LGE imaging in a coronary distribution.

### Estimating pulmonary capillary wedge pressure from the cardiovascular magnetic resonance

Recently, a model to estimate LVFP using CMR‐obtained LVM and LAV has been developed. This model was derived from 835 subjects referred for the investigation of suspected HF who underwent CMR, echocardiography, and invasive assessment.^6^ We utilized the following equation in this study to derive LVFP from LAV and LVM.

$$\mathrm{CMR}-\text{derived LVFP}=6.1352+\left(0.07204\times \mathrm{LAV}\right)+\left(0.02256\times \mathrm{LVM}\right)$$

### Statistical analysis

Continuous variables are reported as mean ± standard deviation and compared using two‐sample independent *t*‐tests. Categorical variables are reported as frequencies and percentages and compared using the *χ* ^2^ statistic. The optimum discriminatory threshold for LVFP in our cohort was determined using the C‐statistics Youden index.

The relationship between all clinically relevant CMR variables against HF hospitalization and MACE was explored using a univariable Cox proportional hazard model analysis. The optimum threshold for pulmonary capillary wedge pressure (PCWP) was derived using C‐statistics. Similarly, Kaplan–Meier survival curve analysis was undertaken for the PCWP cut‐off from C‐statistics for both HF hospitalization‐ and MACE‐free probabilities. Cox proportional hazard model was used for multivariable prognosis analysis. In the multivariable model, only clinically relevant CMR parameters were entered and were kept to a minimum by checking collinearity. For univariable and multivariable models, continuous variables were inputted as *z*‐scores to allow for comparison across different scales.

Statistical analysis was performed in SPSS Version 22 (IBM, Chicago, IL, USA). All statistical tests were two‐tailed, with a *P*‐value of <0.05 deemed significant.

## Results

### Study population

**Figure 1:**
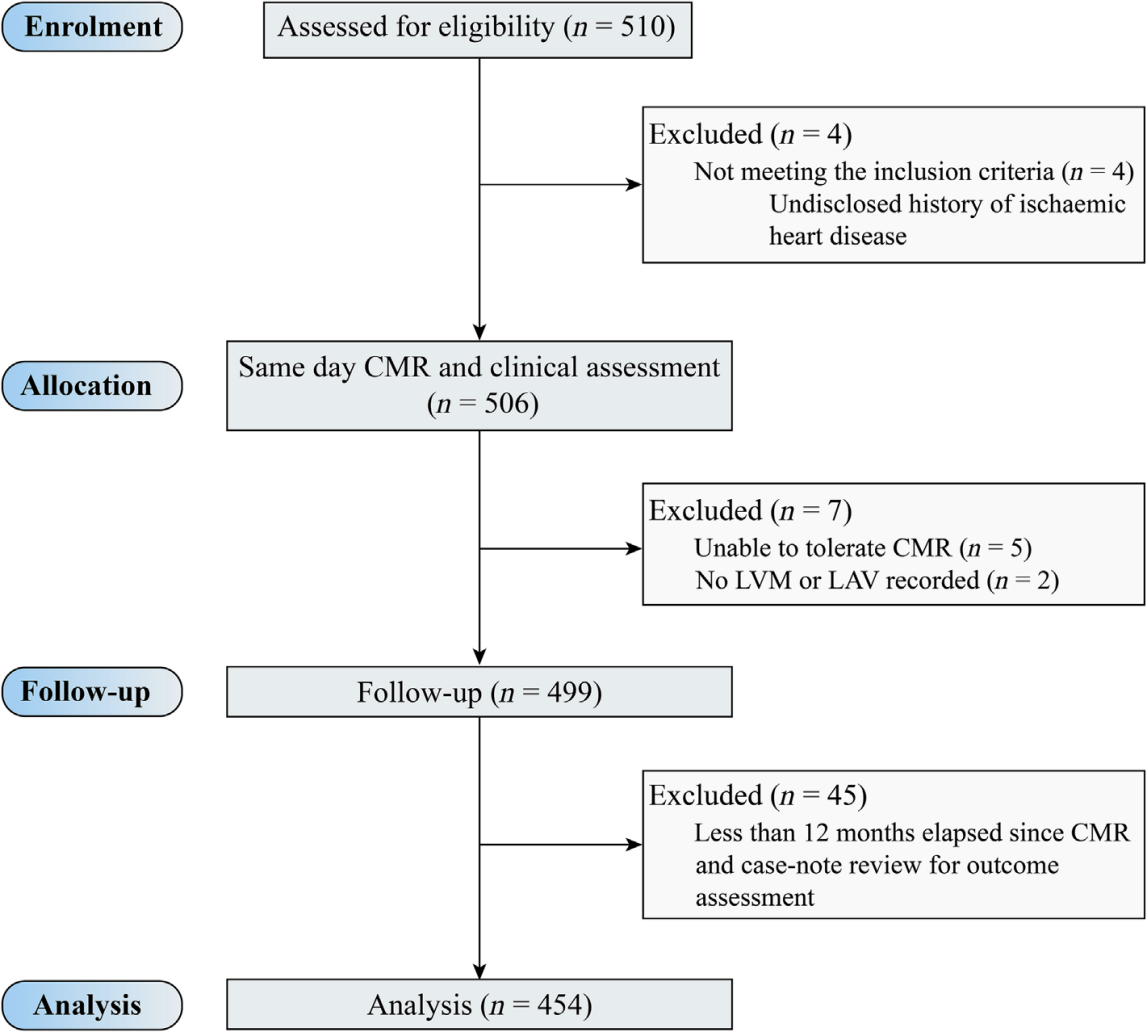
CONSORT diagram. CMR, cardiovascular magnetic resonance; LAV, left atrial volume; LVM, left ventricular mass.

In this study, 510 participants were enrolled between February 2018 and January 2020, with 454 having at least a 12 month follow‐up (*Figure* *1*). The median follow‐up period was 2.9 years. The aetiology of HF within the study population was non‐ischaemic cardiomyopathy (*n* = 363, 79.7%), ischaemic cardiomyopathy (*n* = 88, 19.4%), and cardiac amyloid (*n* = 4, 0.9%). The median interval between echocardiography and CMR was 89 days. The Youden index cut‐off for PCWP (CMR‐derived LVFP) was >16.2 mmHg for MACE [area under the curve 0.63, 95% confidence interval (CI) 0.59–0.68; *P* = 0.0009].

The mean age of the study population was 62 ± 13 years, 64% were male (*n* = 291), and 30% had CMR‐derived LVFP > 16.2 mmHg (*n* = 135). Participants with raised CMR‐derived LVFP were older (64 ± 12 vs. 62 ± 13 years, *P* = 0.009) and more likely to be male (82% vs. 56%, *P* < 0.0001) than those with a normal LVFP (*Table* *1*). Systolic blood pressure was comparable in participants with raised and normal LVFP; however, diastolic blood pressure was higher in participants with raised CMR‐derived LVFP (77 ± 11 vs. 74 ± 10 mmHg, *P* = 0.004). The incidence of atrial fibrillation was higher in participants with raised CMR‐derived LVFP (53% vs. 29%, *P* < 0.0001). HF patients with raised CMR‐derived LVFP were more likely to be receiving diuretic therapy (61% vs. 36%, *P* < 0.0001) and aldosterone‐receptor antagonists (39% vs. 27%, *P* = 0.02). Other than left ventricular (LV) stroke volume, ischaemic scar on LGE, and inducible ischaemia on perfusion, all other LV and right ventricular (RV) volumetric parameters significantly differed between raised and normal CMR‐derived LVFP HF patients (*Table* *1*). LVEF and RVEF were lower in participants with raised CMR‐derived LVFP compared with participants with normal LVFP. NT‐proBNP was significantly higher in participants with raised CMR‐derived LVFP (2154 vs. 990 pg/mL, *P* < 0.001). Between echocardiographic and CMR assessments of EF, 159 participants (35.0%) had transitioned from EF < 40% (echocardiography) to an EF ≥ 40% (CMR).

**Table 1:** Baseline characteristics

Clinical parameters	All subjects (*n* = 454)	LVFP ≤ 16.2 mmHg (*n* = 319)	LVFP > 16.2 mmHg (*n* = 135)	*P*‐value
Age (years)	62.4 ± 12.6.2	61.8 ± 12.9	63.9 ± 12.0	0.009
Gender (male)	291 (64.1)	180 (56.4)	111 (82.2)	<0.0001
Body mass index (kg/m^2^)	30.1 ± 21.0	30.0 ± 24.6	30.3 ± 8.0	0.85
Heart rate (b.p.m.)	70.8 ± 14.5	70.4 ± 14.3	72.0 ± 15.0	0.27
Systolic blood pressure (mmHg)	123.6 ± 19.8	123.3 ± 18.9	124.5 ± 22.0	0.60
Diastolic blood pressure (mmHg)	75.0 ± 10.5	74.0 ± 10.3	77.2 ± 10.8	0.004
**Comorbidities**				
Diabetes mellitus, *n* (%)	70 (15.5)	52 (16.4)	18 (13.3)	0.04
Hypertension, *n* (%)	204 (45.0)	136 (42.8)	68 (50.4)	0.14
Hypercholesterolaemia, *n* (%)	112 (24.7)	79 (24.8)	33 (24.4)	0.93
Cerebrovascular event, *n* (%)	56 (12.4)	40 (14.4)	16 (11.9)	0.83
Atrial fibrillation, *n* (%)	165 (36.5)	93 (29.3)	72 (53.3)	<0.0001
**Smoking**				
Current smoker, *n* (%)	83 (18.3)	60 (18.8)	23 (17.0)	0.69
Ex‐smoker, *n* (%)	160 (35.2)	115 (36.1)	45 (33.3)	
**Medications**				
Antiplatelets, *n* (%)	89 (19.6)	67 (21.3)	22 (16.3)	0.23
Beta‐blocker, *n* (%)	364 (80)	249 (79.0)	115 (85.2)	0.13
Statin, *n* (%)	192 (80.2)	135 (42.9)	57 (42.2)	0.90
ACE‐I/ARB, *n* (%)	376 (82.8)	263 (83.5)	113 (83.7)	0.96
Sacubitril/valsartan, *n* (%)	16 (3.5)	9 (2.8)	7 (5.2)	0.22
Aldosterone‐receptor antagonist, *n* (%)	137 (30.2)	85 (27.0)	52 (38.5)	0.02
Diuretic, *n* (%)	196 (43.2)	114 (36.2)	82 (60.7)	<0.0001
Oral anti‐glycaemic agent, *n* (%)	48 (10.6)	34 (10.8)	14 (10.4)	0.89
Oral anticoagulant, *n* (%)	153 (33.7)	92 (29.2)	61 (45.2)	0.001
**Heart failure phenotype on CMR**				
**Clinical CMR parameters**				
Left ventricular end‐diastolic volume (mL)	213.7 ± 71.7	192.1 ± 55.1	266.2 ± 79.4	<0.0001
Left ventricular end‐systolic volume (mL)	133.4 ± 68.5	113.7 ± 52.6	181.4 ± 78.0	<0.0001
Left ventricular stroke volume (mL)	80.4 ± 26.1	79.1 ± 22.8	84.8 ± 33.1	0.07
Left ventricular ejection fraction	40.2 ± 12.9	42.8 ± 11.9	33.7 ± 12.9	<0.0001
Left ventricular ejection fraction < 40%, *n* (%)	214 (47.1)	127 (40.0)	87 (64.4)	<0.0001
Left ventricular ejection fraction ≥ 40%, *n* (%)	240 (52.9)	192 (60.0)	48 (35.6)	<0.0001
Left ventricular mass (g)	132.5 ± 40.9	121.1 ± 33.7	158.8 ± 44.5	<0.0001
Right ventricular end‐diastolic volume (mL)	151.2 ± 50.6	135.8 ± 37.0	187.8 ± 59.4	<0.0001
Right ventricular end‐systolic volume (mL)	79.2 ± 40.3	66.1 ± 26.5	109.7 ± 50.1	<0.0001
Right ventricular stroke volume (mL)	73.5 ± 26.7	70.1 ± 23.5	78.1 ± 30.3	0.007
Right ventricular ejection fraction (%)	49.0 ± 14.1	51.7 ± 13.6	42.7 ± 13.1	<0.0001
Left atrial volume (mL)	82.1 ± 37.8	61.8 ± 18.5	129.5 ± 28.1	<0.0001
Ischaemic LGE, *n* (%)	82 (18.1)	51 (16.0)	31 (23.0)	0.08
Inducible ischaemia, *n* (%)	25 (5.5)	19 (6.0)	6 (4.4)	0.52
**Cardiac biomarkers**				
NT‐proBNP (pg/mL)	1320 ± 1954	990 ± 845	2154 ± 1167	<0.0001

ACE‐I, angiotensin‐converting enzyme inhibitor; ARB, angiotensin receptor blocker; CMR, cardiovascular magnetic resonance; LGE, late gadolinium enhancement; LVFP, left ventricular filling pressure; NT‐proBNP, N‐terminal pro‐B‐type natriuretic peptide.

### Cardiovascular magnetic resonance‐derived left ventricular filling pressure and symptoms of heart failure

**Figure 2:**
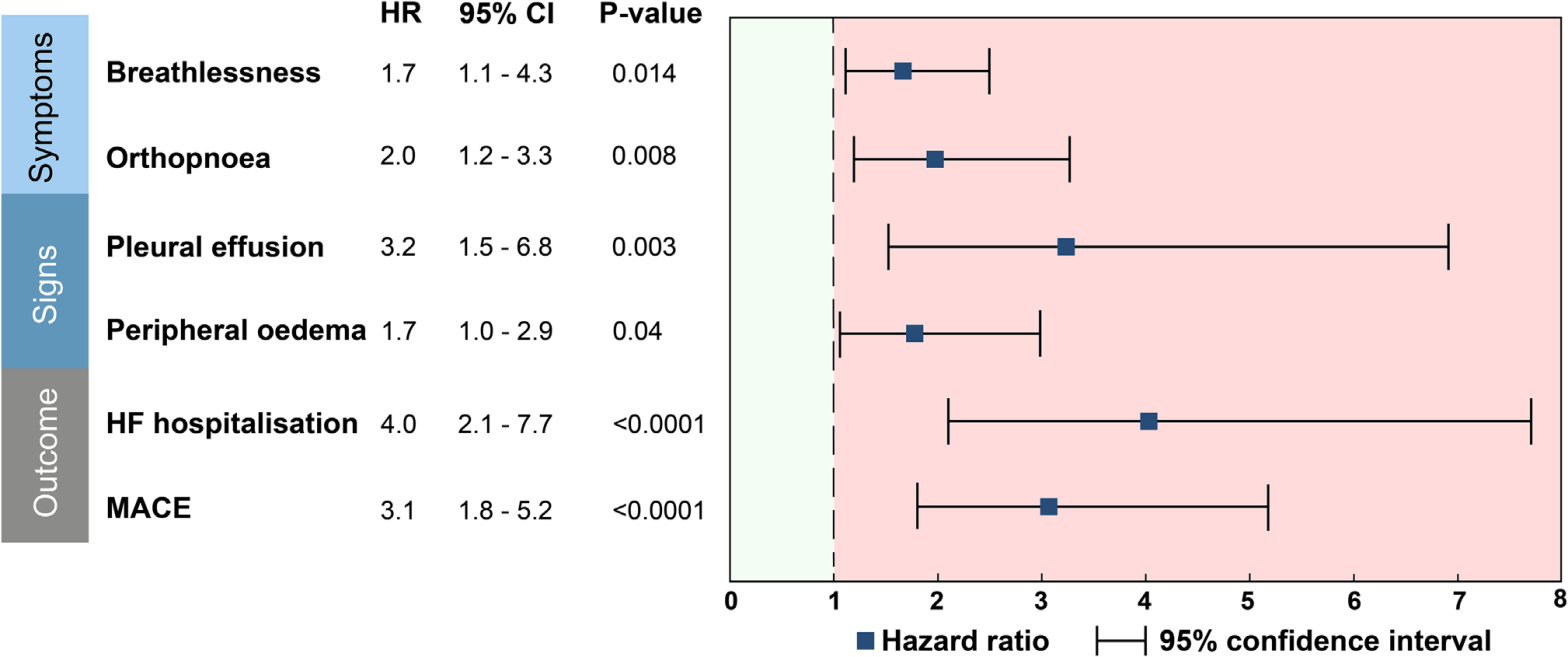
Forest plots demonstrating the association of cardiovascular magnetic resonance‐derived raised left ventricular filling pressure to symptoms and signs of heart failure (HF) and prognosis. CI, confidence interval; HR, hazard ratio; MACE, major adverse cardiovascular events.

Participants with raised filling pressure were more likely to be breathless [hazard ratio (HR) 1.7, 95% CI 1.1–4.3; *P* = 0.01] and suffer from orthopnoea (HR 2.0, 95% CI 1.2–3.3; *P* = 0.008) (*Figure* *2*). However, raised LVFP was not associated with NYHA HF functional class ≥ 2 (*P* = 0.17).

### Cardiovascular magnetic resonance‐derived left ventricular filling pressure and signs of heart failure

Participants with raised filling pressure were more likely to suffer from lower limb pitting oedema (HR 1.7, 95% CI 1.0–2.9; *P* = 0.04) and have pleural effusions (HR 3.2, 95% CI 1.5–6.8; *P* = 0.003) (*Figure* *2*).

### Univariable Cox proportional hazard analysis for heart failure hospitalization and major adverse cardiovascular events

Forty‐two participants (9.3%) had MACE. In those with a normal CMR‐derived LVFP, 31 participants had MACE (9.7%). In those with a raised CMR‐derived LVFP, 11 participants had MACE (8.1%).

Thirty‐eight participants had HF hospitalization. In those with a normal CMR‐derived LVFP, 15 had an HF hospitalization (4.7%). In those with a raised CMR‐derived LVFP, 23 participants had an HF hospitalization (17.0%).

In the univariable Cox proportional hazard analysis for HF hospitalizations and MACE, 14 variables were assessed (*Table* *2*). All variables except right ventricular stroke volume (RVSV), inducible ischaemia, and NT‐proBNP were significantly associated with HF hospitalization. All variables except RVSV and inducible ischaemia were significantly associated with MACE. Supplementary analysis of LVEF dichotomized at 40% is described in Supporting Information, *Table* *S1*.

**Table 2:** Univariable analysis for heart failure hospitalizations and major adverse cardiovascular events

Covariate	Beta	SE	HR	95% CI	*P*‐value
**Heart failure hospitalization**					
Age	0.35	0.19	1.41	0.98–2.03	0.06
Gender	−0.21	0.37	0.81	0.40–1.65	0.6
Left ventricular end‐diastolic volume	0.39	0.14	1.47	1.12–1.94	0.006
Left ventricular end‐systolic volume	0.48	0.13	1.62	1.26–2.09	0.0002
Left ventricular stroke volume	−0.48	0.20	0.62	0.42–0.93	0.02
Left ventricular ejection fraction	−0.58	0.17	0.56	0.40–0.79	0.0008
Left ventricular mass	0.39	0.15	1.47	1.11–1.95	0.008
Right ventricular end‐diastolic volume	0.39	0.12	1.47	1.16–1.88	0.002
Right ventricular end‐systolic volume	0.49	0.11	1.63	1.30–2.03	<0.0001
Right ventricular stroke volume	−0.08	0.17	0.93	0.66–1.29	0.9
Right ventricular ejection fraction	−0.26	0.10	0.77	0.63–0.94	0.009
Left atrial volume	0.37	0.14	1.45	1.11–1.89	0.006
Ischaemic LGE	0.89	0.34	2.40	1.24–4.74	0.01
Inducible ischaemia	−0.18	0.73	0.84	0.20–3.49	0.8
NT‐proBNP > 125 pg/mL	1.96	1.01	7.1	0.97–52.16	0.06
CMR‐derived LVFP > 16.2 mmHg	1.4	0.35	4.02	2.09–7.70	<0.0001
**MACE**					
Age	0.53	0.16	1.71	1.24–2.37	0.001
Gender	−0.38	0.32	0.68	0.37–1.26	0.2
Left ventricular end‐diastolic volume	0.30	0.12	1.35	1.07–1.71	0.01
Left ventricular end‐systolic volume	0.41	0.11	1.51	1.21–1.87	0.0002
Left ventricular stroke volume	−0.33	0.16	0.72	0.52–0.98	0.04
Left ventricular ejection fraction	−0.54	0.14	0.58	0.44–0.77	0.0002
Left ventricular mass	0.32	0.12	1.38	1.08–1.75	0.0009
Right ventricular end‐diastolic volume	0.25	0.12	1.28	1.02–1.60	0.03
Right ventricular end‐systolic volume	0.39	0.10	1.47	1.20–1.80	0.0002
Right ventricular stroke volume	−0.16	0.14	0.86	0.65–1.12	0.3
Right ventricular ejection fraction	−0.23	0.09	0.79	0.67–0.94	0.007
Left atrial volume	0.29	0.12	1.34	1.06–1.68	0.01
Ischaemic LGE	0.91	0.28	2.49	1.43–4.32	0.001
Inducible ischaemia	0.72	0.43	2.06	0.88–4.81	0.09
NT‐proBNP > 125 pg/mL	2.40	1.01	11.00	1.15–79.9	0.02
CMR‐derived LVFP > 16.2 mmHg	1.11	0.27	3.06	1.81–5.17	<0.0001

CI, confidence interval; CMR, cardiovascular magnetic resonance; HR, hazard ratio; LGE, late gadolinium enhancement; LVFP, left ventricular filling pressure; MACE, major adverse cardiovascular events; NT‐proBNP, N‐terminal pro‐B‐type natriuretic peptide.

**Figure 3:**
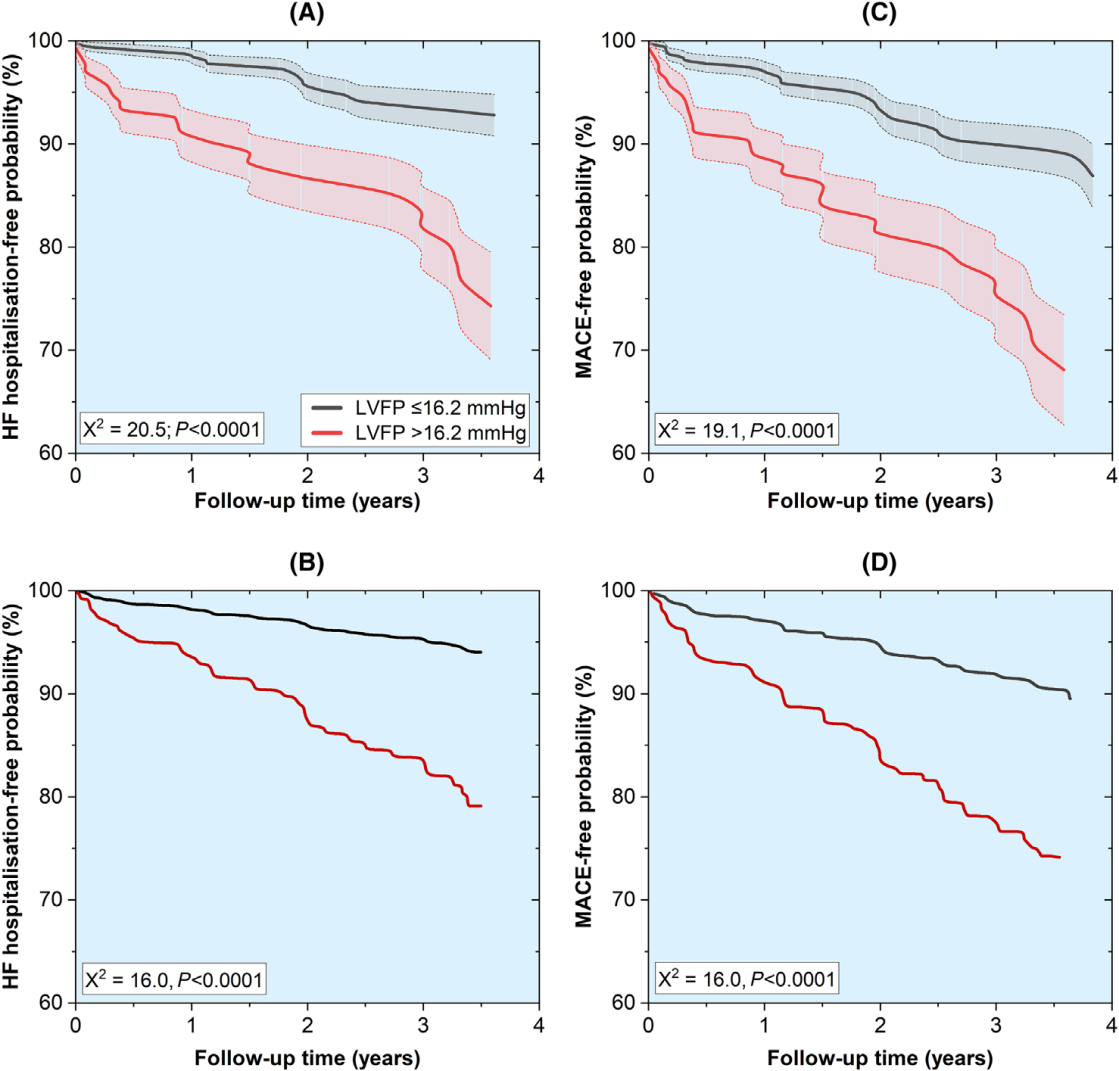
Kaplan–Meier curves. (A) Compared with subjects with normal left ventricular filling pressure (LVFP), those with elevated LVFP had a lower probability of avoiding hospitalization due to heart failure (HF). (B) HF hospitalization‐free probability remains lower in subjects with raised LVFP even after adjusting for the ischaemic scar on cardiovascular magnetic resonance (CMR). (C) Major adverse cardiovascular events (MACE)‐free probability was lower in subjects with raised LVFP. (D) The MACE‐free probability remains lower in subjects with raised LVFP even after adjusting for the ischaemic scar on CMR.

If participants had raised LVFP derived by CMR, they had four times the risk of admission with decompensated HF (HR 4.0, 95% CI 2.1–7.7; *P* < 0.001) (*Table* *2* and *Figure* *2*). In Kaplan–Meier analysis, participants with normal LVFP by CMR had a 7% probability of requiring admission to the hospital over the 2.9 year follow‐up period (93% HF hospitalization‐free probability). In participants with raised CMR‐derived LVFP, the probability of decompensating with HF and requiring hospitalization was 25% (75% HF hospitalization‐free probability) for the same follow‐up period (*χ* ^2^ = 20.5, *P* < 0.0001) (*Figure* *3A*).

If participants had raised LVFP derived by CMR, they had three times the risk of MACE (HR 3.1, 95% CI 1.8–5.2; *P* < 0.001) (*Table* *2* and *Figure* *2*). In Kaplan–Meier survival analysis, participants with normal LVFP by CMR had an 11% probability of MACE (89% MACE‐free probability), and those with raised LVFP had a 32% probability of MACE (68% MACE‐free probability) over the 2.9 year follow‐up period (*χ* ^2^ = 19.1, *P* < 0.0001) (*Figure* *3B*).

Supplementary Kaplan–Meier analysis stratified by LVEF (<40% and ≥40%) suggests that the relationship between raised LVFP and HF hospitalization‐ and MACE‐free probabilities is sustained, regardless of EF (Supporting Information, *Figure* *S1*).

### Multivariable Cox proportional hazard analysis for heart failure hospitalization and major adverse cardiovascular events

After removing variables with shared clinical collinearity, four variables were entered into multivariable Cox regression analysis for HF hospitalization. These were CMR‐derived LVFP > 16.2 mmHg, the presence of ischaemic scar, LVEF, and RVEF. In the stepwise model, two CMR metrics were independently associated with HF hospitalization: CMR‐derived LVFP > 16.2 mmHg (HR 3.80, 95% CI 1.97–7.30; *P* = 0.0001) and the presence of ischaemic scar (HR 2.16, 95% CI 1.10–4.23; *P* = 0.025) (Supporting Information, *Table* *S2*). Adjusted Kaplan–Meier survival analysis for ischaemic scar demonstrated that CMR‐derived LVFP remained a strong predictor for HF hospitalization (*Figure* *3C*).

For MACE, CMR‐derived LVFP > 16.2 mmHg, the presence of ischaemic scar, LVEF, RVEF, and NT‐proBNP > 125 pg/mL were entered in the stepwise multivariable Cox regression analysis. The same two CMR variables demonstrated an independent association to MACE: CMR‐derived LVFP > 16.2 mmHg (HR 2.97, 95% CI 1.74–5.06; *P* = 0.0001) and the presence of ischaemic scar (HR 2.30, 95% CI 1.32–4.02; *P* = 0.003) (Supporting Information, *Table* *S3*). Adjusted Kaplan–Meier survival analysis for ischaemic scar demonstrated that CMR‐derived LVFP remained a strong predictor for MACE (*Figure* *3D*).

**Figure 4:**
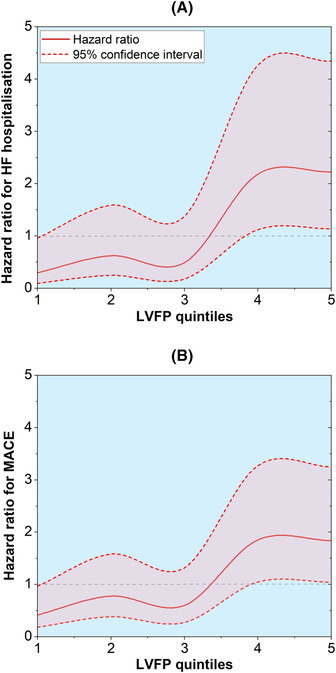
Hazard ratio plots in different quintiles of cardiovascular magnetic resonance (CMR)‐derived left ventricular filling pressure (LVFP). (A) Heart failure (HF) hospitalization risk in different quintiles of CMR‐derived pulmonary capillary wedge pressure. (B) Major adverse cardiovascular events (MACE) risk in different quintiles of CMR‐derived LVFP.

Hazard plots demonstrated that the lowest quintile of CMR‐derived LVFP (median LVFP of 11 mmHg) was associated with a reduced hazard for HF hospitalization (*Figure* *4*). Quintiles four (median LVFP of 16 mmHg) and five (median LVFP of 20 mmHg) were significantly associated with an increased hazard for HF hospitalization and MACE (Supporting Information, *Table* *S4*).

## Discussion

CMR‐derived LVFP has been previously validated in a large cohort of suspected HF patients in a single‐centre study.^6^ In this study, we demonstrate that CMR‐derived LVFP can predict outcomes in people with a recent HF diagnosis. This study also provides reassuring external validation of the CMR‐derived LVFP model. Moreover, in our clinically well‐phenotyped HF patients, we demonstrate how CMR‐derived LVFP is associated with symptoms (breathlessness and orthopnoea) and signs of HF (lower limb oedema and pleural effusion). Most importantly, we further demonstrate how CMR‐derived raised LVFP is associated with a four‐fold risk of decompensation from HF requiring hospitalization and a three‐fold risk of composite MACE.

LVEF and the presence of ischaemic scar are two of the strongest CMR predictors of outcomes in people with HF.^10^ We have demonstrated within multivariable modelling that even when LVEF and ischaemic scar are accounted for, CMR‐derived LVFP is associated with MACE and HF hospitalization. While CMR is the gold standard method for assessing LVEF,^2^, ^3^ as a metric, its influence on outcomes in clinical practice is only modest.^11^ The presence of ischaemic scar signifying prior myocardial infarction, recognized or unrecognized (as in this study), is well known to be associated with adverse outcomes.^12^, ^13^ The largest meta‐analysis to date showed that unrecognized ischaemic scar was associated with a significant risk of MACE (HR 3.23, 95% CI 2.10–4.95).^12^ Our findings support the assessment of CMR‐derived LVFP from a standard examination that already includes assessment of both LVEF and ischaemic scar without needing additional sequences.

The current study differs from the large CMR‐derived LVFP study that initially validated CMR against invasive methods.^6^ This study reports completely different clinical outcomes in patients with HF, namely, hospitalization from decompensated HF and MACE, vs. all‐cause mortality as the clinical outcome within the validation study. The findings of this study are more pertinent to HF populations as prevention of HF hospitalization remains one of the key clinical outcome targets in HF.^14^ In addition, this study tests the clinical value of the CMR‐derived LVFP in an external cohort of HF patients.

An ideal non‐invasive method of LVFP should meet the following conditions for broader clinical adoption and translation: minimal operator dependence; no risk to the person on repeated tests; highly reproducible, non‐inferior to predict clinical outcomes when compared with invasive assessment; and it should permit monitoring of treatment effect(s). CMR‐derived LVFP can address the majority of these conditions. CMR is a safe, non‐invasive test with established precision and reliability in cardiac volumetric assessment.^15^ The CMR‐derived LVFP is dependent on two parameters: maximum LAV and LVM. Both have been shown to have excellent reproducibility in large CMR population‐based studies.^16^, ^17^ It is important to note that, physiologically, LVM is less susceptible to dynamic and acute changes in ventricular loading than LAV, with changes reflecting the chronic burden of raised filling pressures. In contrast, LAV can fluctuate due to variations in pre‐loading conditions. That said, LAV represents the integration of LV diastolic performance over time and is considered a reliable indicator of the severity of diastolic dysfunction.^18^ In HF, as the compliance of the LV deteriorates, the pressure within the left atrium (LA) increases to maintain adequate LV filling, resulting in LA dilatation.^19^ As well as severity, in the absence of atrial fibrillation or mitral valve disease, LA dilatation may provide insight into the chronicity of LV diastolic dysfunction.^20^

Echocardiography is the first‐line non‐invasive method of LVFP assessment. It is versatile and cost‐effective and can estimate LVFP quickly at the bedside. However, the integrated method recommended by the American Society of Echocardiography/European Association of Cardiovascular Imaging in 2016 is complex, and its reliability in informing invasive LVFP remains debatable.^21^ In a large study by Pak *et al*., which recruited 1967 participants, 57% of participants who had raised LVFP by the integrated echocardiography method had normal filling pressure by invasive assessment.^22^ Also, 31% of participants with normal LVFP by echocardiography had raised LVFP by invasive assessment. Even prior studies that evaluated the integrated approach demonstrate modest diagnostic reliability of echocardiography for LVFP.^23^ In our previous work, where we recruited 127 participants who underwent CMR, echocardiography, and invasive assessment, echocardiography‐derived LVFP was indeterminate in 49% of participants.^6^ And, of those participants where echocardiography was non‐diagnostic (indeterminate or incorrect diagnosis), CMR was able to correctly reclassify participants to either ‘normal’ or ‘raised’ LVFP in 71% cases. Therefore, resting CMR‐derived LVFP could provide complementary diagnostic assessment in people with equivocal LVFP by echocardiography.

This study has several limitations. This is a single‐centre observational study of participants with a recent diagnosis of HF. Participants had been referred for CMR at different timepoints following diagnosis, meaning optimal medical therapy may not have been achieved. Although all participants had LVEF < 50% on referral echocardiography, 35% of participants had recovered LV function by the time of the CMR (i.e. transitioned from an EF of <40% to ≥40%). Hence, the results of this study cannot be extrapolated to people at index presentation of HF. Because participants recruited to this study were referred for CMR as part of routine clinical practice, referral bias may have potentially excluded frailer people who were not felt suitable to undergo CMR. Neither invasive catheterization nor same‐day echocardiography data were available for these people to compare CMR‐derived LVFP to them for clinical outcomes.

In this study, we show that raised CMR‐derived LVFP is associated with prognostic outcomes, symptoms, and signs of HF. These findings endorse the CMR‐derived LVFP as a marker of impaired LV diastolic function. Echocardiography will always remain the first‐line test for people presenting with HF symptoms. However, the finding of this study supports clinical pathways that include resting CMR imaging as a gatekeeper to further invasive or stress testing in people with equivocal findings by echocardiography. In these patients, CMR‐derived LVFP can inform the risk of decompensation from HF needing hospitalization and the risk of composite MACE. Future studies are needed to investigate the treatment effect of HF pharmacological intervention by CMR‐derived LVFP for informing diagnosis and guiding the treatment of people with HF.

## Conflict of interest

P.G. is a clinical advisor for Pie Medical Imaging and Medis Medical Imaging. S.P. is a consultant to Circle Cardiovascular Imaging. R.T. is an advisor to Panacea Innovation. All other authors have no conflicts of interest to declare.

## Funding

This work was supported by the British Heart Foundation (RG/16/1/32092), the National Institute for Health Research (NIHR‐RP‐R3‐12‐027), and the Wellcome Trust (220703/Z/20/Z and 215799/Z/19/Z). R.T. and C.G.‐C. are supported by an NIHR Academic Clinical Fellowship.

## Supporting information


**Table S1.** Univariable analysis exploring ejection fraction as a covariate and heart failure hospital and MACE as outcomes.
**Figure S1.** Kaplan Meier curves.
**Panel A:** In subjects with an ejection fraction of <40%, the heart failure hospitalisation‐free probability was lower in subjects with raised LVFP than those with normal LVFP.
**Panel B:** In subjects with an ejection fraction of <40%, the MACE‐free probability was lower in subjects with raised LVFP than those with normal LVFP.
**Panel C:** In subjects with an ejection fraction of ≥40%, the heart failure hospitalisation‐free probability remained lower in subjects with raised LVFP than those with normal LVFP.
**Panel D:** In subjects with an ejection fraction of ≥40%, the MACE‐free probability remained lower in subjects with raised LVFP than those with normal LVFP.
**Table S2.** Multivariable analysis exploring the independent prognostic role of CMR‐derived LVFP over ischaemic scar, LVEF and RVEF for HF hospitalisation.
**Table S3.** Multivariable analysis exploring the independent prognostic role of CMR‐derived LVFP over ischaemic scar, LVEF and RVEF for MACE.
**Table S4.** Heart failure hospitalisations and MACE in different quintiles of CMR‐derived LVFP.

## References

[1] Groenewegen A , Rutten FH , Mosterd A , Hoes AW . Epidemiology of heart failure. Eur J Heart Fail 2020;22:1342–1356. doi:10.1002/ejhf.1858 32483830 PMC7540043

[2] Heidenreich PA , Bozkurt B , Aguilar D , Allen LA , Byun JJ , Colvin MM , *et al*. 2022 AHA/ACC/HFSA guideline for the management of heart failure: A report of the American College of Cardiology/American Heart Association Joint Committee on Clinical Practice Guidelines. J Am Coll Cardiol 2022;79:e263–e421. doi:10.1016/j.jacc.2021.12.012 35379503

[3] McDonagh TA , Metra M , Adamo M , Gardner RS , Baumbach A , Böhm M , *et al*. 2021 ESC guidelines for the diagnosis and treatment of acute and chronic heart failure. Eur Heart J 2021;42:3599–3726. doi:10.1093/eurheartj/ehab368 34447992

[4] Marwick TH . Ejection fraction pros and cons: JACC state‐of‐the‐art review. J Am Coll Cardiol 2018;72:2360–2379. doi:10.1016/j.jacc.2018.08.2162 30384893

[5] Pfeffer MA , Shah AM , Borlaug BA . Heart failure with preserved ejection fraction in perspective. Circ Res 2019;124:1598–1617. doi:10.1161/CIRCRESAHA.119.313572 31120821 PMC6534165

[6] Garg P , Gosling R , Swoboda P , Jones R , Rothman A , Wild JM , *et al*. Cardiac magnetic resonance identifies raised left ventricular filling pressure: Prognostic implications. Eur Heart J 2022;43:2511–2522. doi:10.1093/eurheartj/ehac207 35512290 PMC9259376

[7] Baritussio A , Muthurangu V . Cardiovascular magnetic resonance for the assessment of left ventricular filling pressure in heart failure. Eur Heart J 2022;43:2523–2525. doi:10.1093/eurheartj/ehac247 35574820

[8] Human WM . Experimentation: Code of ethics of W.M.A. Br Med J 1964;2:177. doi:10.1136/bmj.2.5402.177 14150898 PMC1816102

[9] Brown LAE , Saunderson CED , Das A , Craven T , Levelt E , Knott KD , *et al*. A comparison of standard and high dose adenosine protocols in routine vasodilator stress cardiovascular magnetic resonance: Dosage affects hyperaemic myocardial blood flow in patients with severe left ventricular systolic impairment. J Cardiovasc Magn Reson 2021;23:37. doi:10.1186/s12968-021-00714-7 33731141 PMC7971951

[10] Klem I , Klein M , Khan M , Yang EY , Nabi F , Ivanov A , *et al*. Relationship of LVEF and myocardial scar to long‐term mortality risk and mode of death in patients with nonischemic cardiomyopathy. Circulation 2021;143:1343–1358. doi:10.1161/CIRCULATIONAHA.120.048477 33478245

[11] Breathett K , Allen LA , Udelson J , Davis G , Bristow M . Changes in left ventricular ejection fraction predict survival and hospitalization in heart failure with reduced ejection fraction. Circ Heart Fail 2016;9:e002962. doi:10.1161/CIRCHEARTFAILURE.115.002962 27656000 PMC5082710

[12] Yang Y , Li W , Zhu H , Pan XF , Hu Y , Arnott C , *et al*. Prognosis of unrecognised myocardial infarction determined by electrocardiography or cardiac magnetic resonance imaging: Systematic review and meta‐analysis. BMJ 2020;369:m1184. doi:10.1136/bmj.m1184 32381490 PMC7203874

[13] Lipinski MJ , McVey CM , Berger JS , Kramer CM , Salerno M . Prognostic value of stress cardiac magnetic resonance imaging in patients with known or suspected coronary artery disease: A systematic review and meta‐analysis. J Am Coll Cardiol 2013;62:826–838. doi:10.1016/j.jacc.2013.03.080 23727209 PMC3863376

[14] Vaduganathan M , Claggett BL , Desai AS , Anker SD , Perrone SV , Janssens S , *et al*. Prior heart failure hospitalization, clinical outcomes, and response to sacubitril/valsartan compared with valsartan in HFpEF. J Am Coll Cardiol 2020;75:245–254. doi:10.1016/j.jacc.2019.11.003 31726194 PMC7983315

[15] Leiner T , Bogaert J , Friedrich MG , Mohiaddin R , Muthurangu V , Myerson S , *et al*. SCMR position paper (2020) on clinical indications for cardiovascular magnetic resonance. J Cardiovasc Magn Reson 2020;22:76. doi:10.1186/s12968-020-00682-4 33161900 PMC7649060

[16] Petersen SE , Matthews PM , Francis JM , Robson MD , Zemrak F , Boubertakh R , *et al*. UK Biobank's cardiovascular magnetic resonance protocol. J Cardiovasc Magn Reson 2015;18:8. doi:10.1186/s12968-016-0227-4 PMC4736703 26830817

[17] Suinesiaputra A , Bluemke DA , Cowan BR , Friedrich MG , Kramer CM , Kwong R , *et al*. Quantification of LV function and mass by cardiovascular magnetic resonance: Multi‐center variability and consensus contours. J Cardiovasc Magn Reson 2015;17:63. doi:10.1186/s12968-015-0170-9 26215273 PMC4517503

[18] Maceira AM , Cosín‐Sales J , Roughton M , Prasad SK , Pennell DJ . Reference left atrial dimensions and volumes by steady state free precession cardiovascular magnetic resonance. J Cardiovasc Magn Reson 2010;12:65. doi:10.1186/1532-429X-12-65 21070636 PMC2994941

[19] Tsang TSM , Barnes ME , Gersh BJ , Bailey KR , Seward JB . Left atrial volume as a morphophysiologic expression of left ventricular diastolic dysfunction and relation to cardiovascular risk burden. Am J Cardiol 2002;90:1284–1289. doi:10.1016/S0002-9149(02)02864-3 12480035

[20] Simek CL , Feldman MD , Haber HL , Wu CC , Jayaweera AR , Kaul S . Relationship between left ventricular wall thickness and left atrial size: Comparison with other measures of diastolic function. J Am Soc Echocardiogr 1995;8:37–47. doi:10.1016/S0894-7317(05)80356-6 7710749

[21] Nagesh SF , Smiseth OA , Appleton CP , Byrd BF , Dokainish H , Edvardsen T , *et al*. Recommendations for the evaluation of left ventricular diastolic function by echocardiography: An update from the American Society of Echocardiography and the European Association of Cardiovascular Imaging. Eur Heart J Cardiovasc Imaging 2016;17:1321–1360. doi:10.1093/ehjci/jew082 27422899

[22] Pak M , Kitai T , Kobori A , Sasaki Y , Okada T , Murai R , *et al*. Diagnostic accuracy of an integrated echocardiographic algorithm to estimate left ventricular filling pressure. J Am Coll Cardiol Img 2022;15:1683–1691. doi:10.1016/j.jcmg.2022.03.022 36202447

[23] Lancellotti P , Galderisi M , Edvardsen T , Donal E , Goliasch G , Cardim N , *et al*. Echo‐Doppler estimation of left ventricular filling pressure: Results of the multicentre EACVI Euro‐Filling study. Eur Heart J Cardiovasc Imaging 2017;18:961–968. doi:10.1093/ehjci/jex067 28444160

