# Supplementary material for: Cardiac magnetic resonance left ventricular filling pressure is linked to symptoms, signs and prognosis in heart failure
Source: ESC Heart Fail. 2023 Aug 19;10(5):3067–76. doi: 10.1002/ehf2.14499 (PMC10567675; doi:10.1002/ehf2.14499)
Supplement: Supplementary file 1 — Table S1. Univariable analysis exploring ejection fraction as a covariate and heart failure hospital and MACE as outcomes. Figure S1. Kaplan Meier curves. Panel A: In subjects with an ejection fraction of <40%, the heart failure hospitalisation‐free probability was lower in subjects with raised LVFP than those with normal LVFP. Panel B: In subjects with an ejection fraction of <40%, the MACE‐free probability was lower in subjects with raised LVFP than those with normal LVFP. Panel C: In subjects with an ejection fraction of ≥40%, the heart failure hospitalisation‐free probability remained lower in subjects with raised LVFP than those with normal LVFP. Panel D: In subjects with an ejection fraction of ≥40%, the MACE‐free probability remained lower in subjects with raised LVFP than those with normal LVFP. Table S2. Multivariable analysis exploring the independent prognostic role of CMR‐derived LVFP over ischaemic scar, LVEF and RVEF for HF hospitalisation. Table S3. Multivariable analysis exploring the independent prognostic role of CMR‐derived LVFP over ischaemic scar, LVEF and RVEF for MACE. Table S4. Heart failure hospitalisations and MACE in different quintiles of CMR‐derived LVFP. [file EHF2-10-3067-s001.docx]

# **Supplementary Materials**

**Supplementary Table 1.** Univariable analysis exploring ejection fraction as a covariate and heart failure hospital and MACE as outcomes

| **Covariate** | **Beta** | **SE** | **HR** | **95% CI** | **P-value** |
| --- | --- | --- | --- | --- | --- |
| **Heart failure hospitalisation** | | | | | |
| Left ventricular ejection fraction | -0.58 | 0.17 | 0.56 | 0.40 – 0.79 | 0.0008 |
| Left ventricular ejection fraction (<40%) | -0.09 | 0.25 | 0.92 | 0.87 – 0.96 | 0.0004 |
| Left ventricular ejection fraction (≥40%) | 0.04 | 0.04 | 1.04 | 0.96 – 1.13 | 0.3 |
| **MACE** | | | | | |
| Left ventricular ejection fraction | -0.54 | 0.14 | 0.58 | 0.44 – 0.77 | 0.0002 |
| Left ventricular ejection fraction (<40%) | -0.06 | 0.02 | 0.94 | 0.91 – 0.98 | 0.0004 |
| Left ventricular ejection fraction (≥40%) | 0.01 | 0.04 | 1.00 | 0.94 – 1.08 | 0.8 |

**Supplementary Figure 1.** Kaplan Meier curves

**Panel A:** In subjects with an ejection fraction of <40%, the heart failure hospitalisation-free probability was lower in subjects with raised LVFP than those with normal LVFP.

**Panel B:** In subjects with an ejection fraction of <40%, the MACE-free probability was lower in subjects with raised LVFP than those with normal LVFP.

**Panel C:** In subjects with an ejection fraction of ≥40%, the heart failure hospitalisation-free probability remained lower in subjects with raised LVFP than those with normal LVFP.

**Panel D:** In subjects with an ejection fraction of ≥40%, the MACE-free probability remained lower in subjects with raised LVFP than those with normal LVFP.


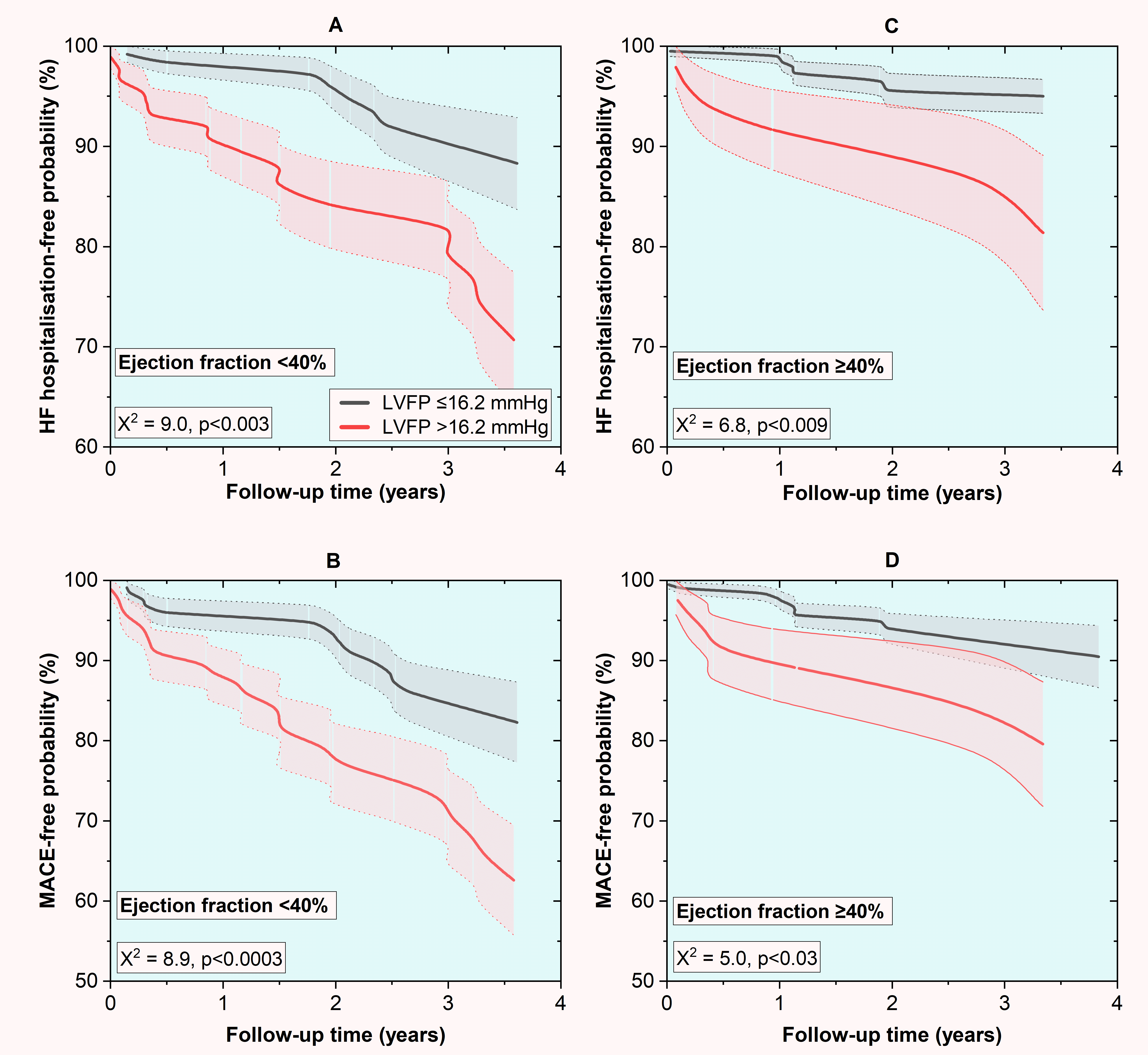


**Supplementary Table 2.** Multivariable analysis exploring the independent prognostic role of CMR-derived LVFP over ischaemic scar, LVEF and RVEF for HF hospitalisation

| **Multivariable Cox regression for decompensated HF hospitalisation** | | | | | |
| --- | --- | --- | --- | --- | --- |
| Input variables | LVFP >16.2mmHg, ischaemic scar, LVEF, and RVEF | | | | |
| Method | Stepwise, variable entered if p < 0.05 | | | | |
| **Covariate** | **Beta** | **SE** | **HR** | **95% CI** | **P-value** |
| LVFP >16.2 mmHg | 1.3 | 0.3 | 3.80 | 1.97 - 7.30 | 0.0001 |
| Ischaemic scar | 0.8 | 0.3 | 2.16 | 1.10 - 4.23 | 0.025 |
| Variables out | LVEF and RVEF | | | | |

**Abbreviations**: CI - confidence interval; HF - heart failure; HR - hazard ratio; LVEF - left ventricular ejection fraction; LVFP - left ventricular filling pressure; RVEF - right ventricular ejection fraction; SE - standard error

**Supplementary Table 3.** Multivariable analysis exploring the independent prognostic role of CMR-derived LVFP over ischaemic scar, LVEF and RVEF for MACE

| **Multivariable Cox regression for MACE** | | | | | |
| --- | --- | --- | --- | --- | --- |
| Input variables | LVFP >16.2mmHg, ischaemic scar, LVEF, RVEF, NT-proBNP | | | | |
| Method | Stepwise, variable entered if p < 0.05 | | | | |
| **Covariate** | **Beta** | **SE** | **HR** | **95% CI** | **P-value** |
| LVFP >16.2 mmHg | 1.1 | 0.3 | 2.97 | 1.74 - 5.06 | 0.0001 |
| Ischaemic scar | 0.8 | 0.3 | 2.30 | 1.32 - 4.02 | 0.003 |
| Variables out | LVEF, RVEF and NT-proBNP >125 pg/mL | | | | |

## **Supplementary Table 4.** Heart failure hospitalisations and MACE in different quintiles of CMR-derived LVFP

| **LVFP quintile** | **Number of patients** | **Median (IQR)** | **Beta** | **SE** | **HR** | **95% CI** | **P-value** |
| --- | --- | --- | --- | --- | --- | --- | --- |
| **Heart failure hospitalisation** | | | | | | | |
| 1 | 91 | 11 (11 - 12) | -1.23 | 0.60 | 0.29 | 0.09 – 0.95 | 0.04 |
| 2 | 91 | 13 (13 - 13) | -0.48 | 0.48 | 0.62 | 0.24 – 1.58 | 0.32 |
| 3 | 90 | 14 (14 - 15) | -0.72 | 0.53 | 0.49 | 0.17 – 1.37 | 0.17 |
| 4 | 91 | 16 (16 - 17) | 0.78 | 0.34 | 2.18 | 1.11 – 4.26 | 0.02 |
| 5 | 91 | 20 (19 - 21) | 0.80 | 0.34 | 2.22 | 1.14 – 4.34 | 0.02 |
| **MACE** | | | | | | | |
| 1 | 91 | 11(11 - 12) | -0.88 | 0.43 | 0.41 | 0.18 – 0.96 | 0.04 |
| 2 | 91 | 13 (13 - 13) | -0.26 | 0.36 | 0.77 | 0.38 – 1.58 | 0.48 |
| 3 | 90 | 14 (14 - 15) | -0.52 | 0.40 | 0.60 | 0.27 – 1.31 | 0.20 |
| 4 | 91 | 16 (16 - 17) | 0.61 | 0.29 | 1.85 | 1.04 – 3.26 | 0.04 |
| 5 | 91 | 20 (19 - 21) | 0.61 | 0.29 | 1.84 | 1.04 – 3.25 | 0.04 |

**Abbreviations**: CI - confidence interval; HR - hazard ratio; IQR - interquartile range; SE - standard error
